# Trophic effects of multiple administration of mesenchymal stem cells in children with osteogenesis imperfecta

**DOI:** 10.1002/ctm2.385

**Published:** 2021-04-04

**Authors:** Sowmya Ramesh, Dolly Daniel, Cecilia Götherström, Vrisha Madhuri

**Affiliations:** ^1^ Department of Paediatric Orthopaedics Christian Medical College Vellore Tamil Nadu India; ^2^ Center for Stem Cell Research, a unit of inStem Bengaluru Christian Medical College Vellore Tamil Nadu India; ^3^ Department of Immunohematology and Transfusion Medicine Christian Medical College Vellore Tamil Nadu India; ^4^ Department of Clinical Science, Intervention & Technology Karolinska Institutet Stockholm Sweden

**Keywords:** brittle bone disease, growth chart, intraosseous, stem cell therapy

## Abstract

The safety of mesenchymal stem cell therapy for osteogenesis imperfecta has been demonstrated previously. However, it is unknown how the trophic effects are mediated by stem cells. In the present commentary, we bring to the attention of readers the recent report by Infante et al in the journal of clinical and translational medicine. The TERCELOI clinical trial presented the possible paracrine effect of transplanted MSCs *in vitro* and i*n vivo* using proteomics and transcriptomic analysis. This novel finding adds new knowledge in the field of regenerative medicine. However, the scarcity of solid evidence in growth warrants a more thorough discussion.

## 1 BACKGROUND

Cell therapy for osteogenesis imperfecta has been under investigation in humans since 1999.^1^ Studies so far have focussed mainly on improving the engraftment potential of the infused stem cells after prenatal or postnatal transplantation. Researchers have demonstrated the functional outcome of cell therapies^2^, ^3^ in OI in terms of improved growth velocity and fracture rate, but there exists a gap‐in‐knowledge regarding the paracrine effects mediated by multiple administration of mesenchymal stem cells (MSCs) in patients with OI.

The therapeutic efficacy of MSCs in many clinical trials varying from regenerative medicine to immunomodulation has shown inconsistent results. One of the possible reasons is the batch to batch variation in the *in vitro* efficacy and quality of cells arising due to donor characteristics.^4^ This may eventually limit its engraftment potential and effect upon transplantation and makes it difficult to interpret the clinical outcomes. More recently, to overcome this limitation, the possibility of using MSC derived from induced pluripotent stem cells has been advocated for the ease of providing large batches of cell products with the same quality. However, their therapeutic use is still preliminary.^5^

Since it appears that the outcome of MSC transplantation in children with OI is transient, there is a need to find out more about the cell‐mediated effects in improving the disease phenotype if this modality is to become a standard of care for the treatment of OI. Therefore, it is appropriate to go back to the bench with the knowledge obtained from the bedside during clinical trials.

## 2 COMMENTARY

We read with great interest the recent research article by Infante and colleagues,^6^ where they have addressed the paracrine effect in their TERCELOI clinical trial, using sera from patients collected before, during, and after the MSC administration. In their clinical trial on two children with severe OI who received multiple MSC administrations isolated from HLA‐matched siblings, and who were followed for over 2 years, they performed extensive proteomics and transcriptomics analysis to study the paracrine effects of the infused MSCs. They showed increased expression of proteins governed by TGF‐beta and BMP signaling pathways to a varying extent with upregulated signaling molecules of osteoprogenitors, collagen‐binding, and extracellular matrices. *In vitro* osteoblastic effects of sera obtained from MSC‐infused patients on MSCs derived from OI patients provided a novel model to study the paracrine effects in patients. Mechanistic studies demonstrated a pro‐osteogenic response from sera of the two children with OI treated with MSC. These results are consistent with the possibility that indeed paracrine activity contributed to an overall improvement in the quality of life in the treated children. However, there are some areas in which their results lead to more thought‐provoking questions.

The authors in TERCELOI trial state that there is no previous *in vivo* study demonstrating the trophic effects induced in OI patients. Previous studies, Horwitz et al.^3^ and Götherström et al.^7^ have demonstrated and noted an increase in growth velocity as one of the significant demonstrable trophic effects after intravenous either postnatal or prenatal cell (bone marrow or fetal liver‐derived MSC) administration. Horwitz reported an acceleration of growth velocity of 60% to 94% of the predicted median over the 6 months immediately preceding the cell administration. Similarly, the TERCELOI study could have been supported by serial height and weight data before and after the injections, as a beneficial clinical trophic effect of the MSCs.

We have an ongoing Boost to Brittle Bones clinical trial (BOOST2B‐ NCT04623606) in Vellore, India, where fetal liver‐derived MSCs manufactured in Sweden are administrated to children with severe OI. The BOOST2B and TERCELOI trials have similarities and differences in their study design. The TERCELOI trial has addressed the effect in older children (aged 6 and 8 years) who received five doses of bone marrow derived‐MSCs (4 × 10^6^ cells/kg) from HLA‐matched siblings at 5–6 months interval. While both trials address the effect of multiple intravenous doses of MSC in OI children with COL1A1/COL1A2 abnormality, the BOOST2B trial, in addition to four doses of intravenous MSC (3 × 10^6^ cells/kg), examines intraosseous injection (0.4 × 10^6^ cells/kg) in all four lower limb long bones. Furthermore, in the BOOST2B trial, the MSC are derived from first trimester fetal liver tissue (the blood forming organ during the first trimester), there is no HLA matching and the four doses are administered at fours month intervals to children with an age of 1–4 years. Lastly, unlike TERCELOI, the BOOST2B trial requires initiation of bisphosphonates before MSC administration.

**FIGURE 1:**
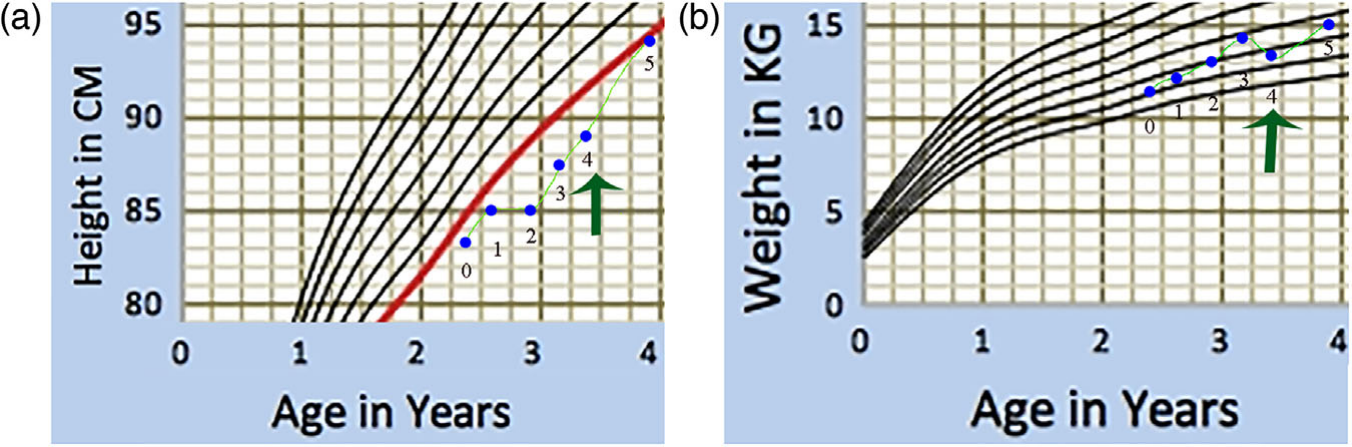
Growth chart of a male child with severe type III OI. The increase in (A) height and (B) weight from visit 0 to 5 are plotted (blue) on the Indian Academy of Paediatrics and WHO growth chart. The red line indicates the third percentile, while the remaining curves correspond to 10th, 25th, 50th, 75th, 90th, and 97th percentile. The visit 4 indicates the first administration of MSCs (green arrow). Visit 5 refers to 4 months after first dose of MSC administration

The first case in BOOST2B trial showed an increase in the growth rate from 0.92% to 1.4% per month, allowing the child to jump to the higher third percentile of growth charts (Figure 1) four months after the first intravenous and intraosseous dose of complete HLA mismatched (0/6 loci) MSCs. The recipient was diagnosed with severe OI caused by a glycine substitution mutation in the *COL1A1* gene and did not receive any immunosuppression before or after the MSCs were administered. Contrary to the TERCELOI authors' hypothesis that HLA matching is required for multiple MSC administration, the two borderline elevated donor‐specific antibodies (MFI 834 and 843) against HLA and other non‐donor specific antibodies prior to the MSC administration were lowered to insignificant levels after the first MSC administration in the described child. To gain further insight, we in the BOOST2B trial performed the single antigen bead assays following incubation of the sera collected before the first administration along with donor MSCs for different periods. The marked apparent decline in donor‐specific antibodies raises the possibility that this could be secondary to a phenomenon of adsorption of antibodies by MSCs thus contributing to the patient's observed phenomenon. Although both trials have used different sources of MSCs the HLA would remain the same irrespective of its source (eg, bone marrow or fetal liver) as the HLA loci being tested for are identical. However, the level of HLA antigen expression may vary between fetal and adult MSCs^8^ and that the HLA expression is lower on the former.^9^ None of the trials included the use of immunosupressants.

Next, the radiographic data to assess trabecular bone microstructure in the TERCELOI study uses a technique that is not described in the supporting information and is not validated or ideal for pediatric osteoporotic condition. The references provided are related to sheep tibia, and adult female femur bone with osteoporosis^10^, ^11^, ^12^ and are not validated for pediatric radiographs of lower femur considering their dynamic Z‐scores. The dual‐energy X‐ray absorptiometry (DEXA) described by Elizabeth Szalay for the distal femur, and lumbar spine could have provided a more appropriate assessment of the lower limb trabecular bone changes in the pediatric age group.^13^, ^14^, ^15^ The lower femur DEXA has a significant advantage in these children as it is easier to position and assess than the distal radius or neck of the femur. Also, in a single image, it is possible to depict the density of the metaphyseal cancellous (newer) bone, the transitional, and the cortical (older) bone.^13^

**FIGURE 2:**
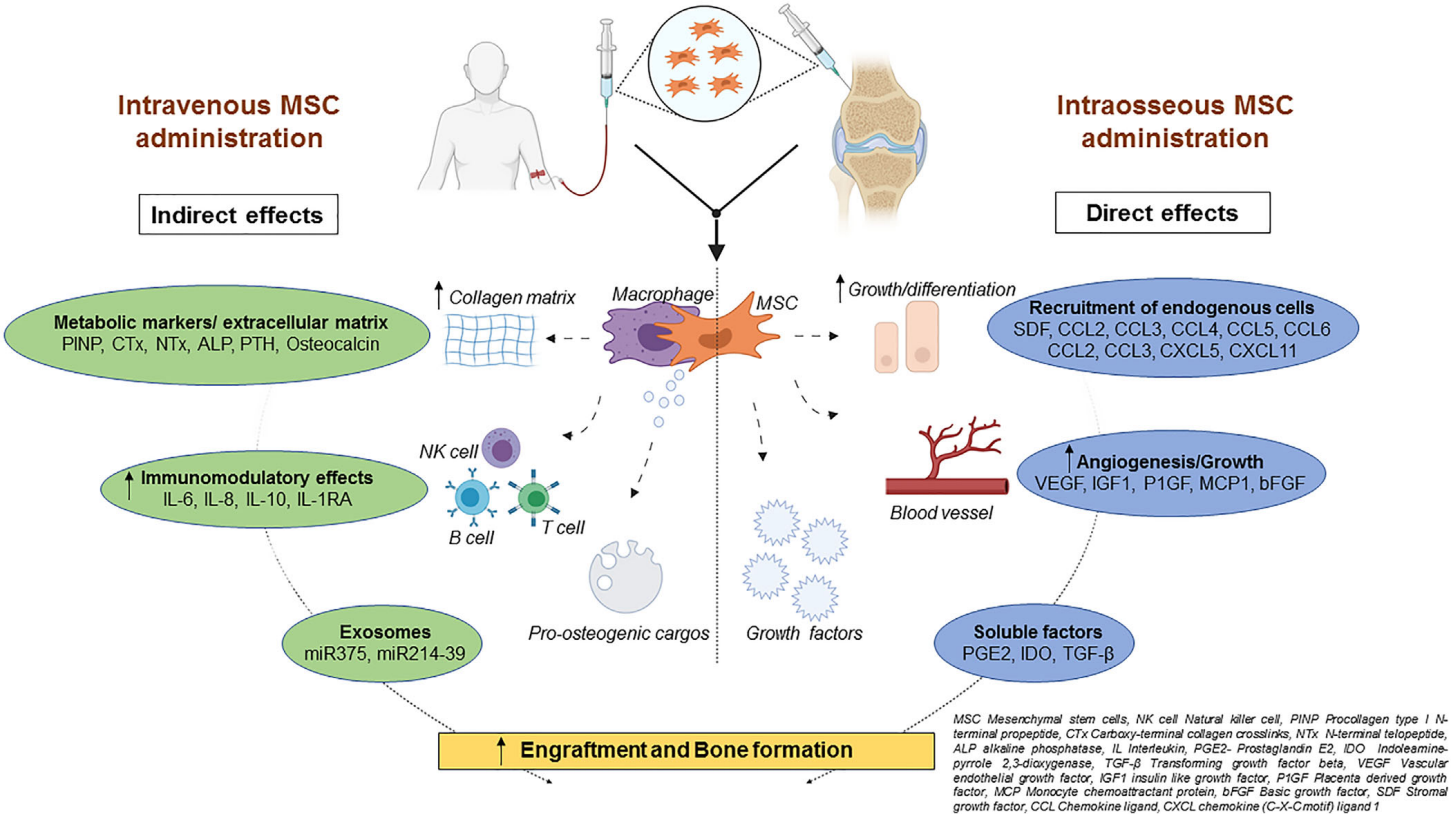
A figure showing the hypothetical *in vivo* molecular mechanism of mesenchymal stem cells and its action on various cellular functions after administration based on the literature^16^, ^17^, ^18^ and the TERCELOI^6^ trial data. Intravenous and intraosseous MSC administration could have direct or indirect effects on increased cell engraftment and bone formation that are mediated by various pathways as stated along with their key players

Finally, the *in vivo* pro‐osteogenic stimulus assessment could be biochemically supported by investigating the bone metabolism by studying changes in bone formation markers such as serum blood specific alkaline phosphatase, osteocalcin, procollagen type I pro‐peptide concentrations; and bone resorption markers such as C‐ terminal telopeptide (CTX), and N‐terminal telopeptide (NTX). Other possible paracrine mechanism that are likely to contribute to the therapeutic effects include exosomes/extracellular vesicles, miRNAs, cytokines, and growth factors that play a critical role in modulating the immune system and take part in the process of differentiation, which are dependent on a pro‐inflammatory environment (Figure 2).

## 3 CONCLUSION

In conclusion, we here provide observation from our ongoing BOOST2B clinical trial to strengthen the recent clinical report of TERCELOI trial where repeated MSC therapy in two pediatric patients with OI was found feasible and safe. It is possible that the use of fetal MSCs and the additional intraosseous dose route could add to the trophic effect as cells administered via this route bypass the pulmonary filtration, facilitate engraftment by reaching their supportive niches, wherein they may proliferate and differentiate. However, the preliminary results of safety from the very inadequate data should be considered with the limitation that there is a theoretical risk of spontaneous transformation and or differentiation of transplanted MSCs into undesirable cell lineages and tissue types *in vivo*.^8^ We believe that evidence for effects of engrafted MSC in OI is still lacking, and this can only be provided by immunohistology of the treated child's bone demonstrating an actual change in the quality of the extracellular matrix, and this may come only with a successful engraftment of non‐OI donor MSCs.
